# Underutilized and undertheorized: the use of hospitalization for ambulatory care sensitive conditions for assessing the extent to which primary healthcare services are meeting needs in British Columbia First Nation communities

**DOI:** 10.1186/s12913-018-3850-y

**Published:** 2019-01-18

**Authors:** Josée G. Lavoie, Sabrina T. Wong, Naser Ibrahim, John D. O’Neil, Michael Green, Amanda Ward

**Affiliations:** 1 Dept of Community Health Sciences, University of Manitoba, #715 – 727 McDermot Avenue, Winnipeg, MB R3E 3P4 Canada; 2 School of Nursing, University of British Columbia, Vancouver, Canada; 3 Ongomiizwin Research, University of Manitoba, Winnipeg, Canada; 4 Simon Fraser University, Burnaby, Canada; 5 Queens University, Kingston, Canada; 6 First Nation Health Authority, Vancouver, Canada

**Keywords:** Avoidable hospitalization, Indigenous populations, Primary care, Rural and remote care

## Abstract

**Background:**

Since the 1960s, the federal government has been providing or funding a selection of community-based primary healthcare (PHC) programs on First Nations reserves. A key question is whether local access to PHC can help address health inequities in First Nations on-reserve communities in British Columbia (BC).

**Objectives:**

This paper examines whether hospitalization for Ambulatory Care Sensitive Conditions (1) can be used as a proxy measure for the organization of PHC in First Nations reserve areas; and (2) is associated with premature mortality rates.

**Methods:**

In this descriptive correlational study, we used administrative data available through Population Data BC, including demographic and ecological information (i.e. geo-codes indicating location of residence). We used two different measures of hospitalization: rates of episodic hospital care and rates of length of stay. We correlated hospitalization rates with premature mortality rates and the level of care available in First Nations communities, which depends on a federal funding formula based upon community size and, more specifically, the level of isolation from a provincial point of care.

**Results:**

First Nations communities in BC that have local 24/7 access to PHC services have similar rates of hospitalization for ACSC to those living in urban centres. This is demonstrated by the similarities in the strengths of the correlation between premature mortality rates and rates of avoidable hospitalization for conditions treatable in a PHC setting. This is not the case for communities served by a Health Centre (weaker correlation) and for communities serviced by a Health Station or with no on-reserve point of care (no correlation).

**Conclusions:**

Improving access to PHC services in First Nations communities can be associated with a significant reduction in avoidable hospitalization and premature mortality rates. The method we tested is an important tool that could serve health care planning decisions in small communities.

## Background

Closing the gap on health and healthcare inequities is an important goal for primary health care (PHC) reforms [1]. One way to redress these inequities is to address the health and healthcare needs of those who experience the worse health outcomes [2] by strengthening the area of PHC. It is well documented that complex morbidities can be both a cause and a consequence of social exclusion [3]. As an example, those living on First Nations reserves in Canada have higher reported rates of avoidable hospitalizations [4], higher premature mortality rates [5], less developed infrastructure (e.g. roads, housing, access to safe drinking water, etc., [6]), and poorer access to responsive primary healthcare (PHC) and effective continuity of care [7, 8]. The 1996 Royal Commission on Aboriginal Peoples’ report [9, 10] and the recent Truth and Reconciliation report [11] documented historical and contemporary instances of systemic and overt discrimination and racism, which perpetuate health inequities, and called for immediate action.

In this paper, we distinguish between the concepts of PHC and primary care. We define PHC as all interventions intended to prevent the onset of disease (nutrition education, for example), to delay their progression (i.e., HbA1c monitoring for diabetic patients**),** and to manage complications (i.e., foot care). Comprehensive PHC includes primary care interventions, which refers to out-patient treatments generally provided by a Family Physician, a Nurse Practitioner, or a nurse with an expanded scope of practice. It further includes efforts to address health inequalities through public health interventions, health promotion and preventative care, patient- and community-centred care, and coordination with related social and health interventions.

Since the late 1960s, Canada has been providing access to healthcare to all Canadians under a single payer system. Co-payments and access fees were made illegal in 1984. In theory, all Canadians can therefore access required care. This is true for First Nations living in urban areas or on parcels of traditional lands called “reserves”, which are federally managed for historical reasons (see [12] for a more comprehensive discussion). While First Nations communities have access to a complement of PHC services funded by the federal government and delivered on reserve by either federal or community employees, years of siloed underfunding [13] and jurisdictional fragmentation [7, 8, 14] have created systemic barriers to accessing a broader complement of responsive PHC than what is accessible locally, as well as barriers to continuity of care to services provided off reserve (Family Physicians, specialists, hospital care, diagnostic care are accessed off-reserve and paid by provincial governments). Previous studies conducted in Manitoba have indicated that First Nations communities with access to a broader complement of PHC delivered on reserve in Nursing Stations (these are facilities where resident nurses with an expanded scope of practice deliver PHC) have lower rates of hospitalization for conditions that are manageable in a PHC setting [4]. In order to examine how well PHC services operating on First Nations reserves in BC are able to meet community needs, indicators from already available longitudinal data sources are needed. In this paper, we examine the utility of using hospitalization for Ambulatory Care Sensitive Conditions (hACSC) as a potential indicator of equitable access to responsive health care.

Inequities in PHC may arise from a lack of care, untimely access to care, unresponsive care, or differential treatment [15], all of which might result in hACSC and/or premature mortality. Since Weissman et al.’s [16] and Billings and colleagues’ [17] seminal papers, the concept of hACSC has gained popularity in higher and increasingly middle income countries as a measure of the performance of the PHC system (see [18] for a review). Billings et al. defined ACSC as, “(t)hose diagnoses for which timely and effective outpatient [primary] care can help to reduce the risks of hospitalization by either preventing the onset of an illness or conditions, controlling an acute episodic illness or conditions, or managing a chronic disease or condition” ([17], p., 163) While many hospitalizations are justified and therefore unavoidable, disproportionate rates of hACSCs could indicate that the PHC system is either:

- inaccessible (geographically or economically);
- ineffective (poor continuity of care, lack of human resources, poor access to diagnosis technologies); or
- unresponsive (poor quality, alternative motivations, discrimination, lack of cultural safe and trauma-informed care).

Past work about the relationship between PHC and hACSCs remains limited and largely undertheorized. Many studies have focused on conceptual work and debates over the definition of ACSC (for examples, [19–25]). Some work has shown that the supply of hospital beds is strongly correlated with hACSC. Other work has addressed the relationship between PHC resourcing and hACSC [26, 27]; both showed a strong negative correlation between the funding of PHC and rates of hACSC. Van Loenen and colleagues’ paper on the organizational aspects of PHC related to avoidable hospitalization for chronic conditions [28] highlighted provider continuity, comprehensiveness, multi-disciplinary care, access, and quality of care (adherence to clinical guidelines) as key factors correlated to lower rates of hACSC. They found mixed results with factors related to the organization of PHC (practice type, size, specific services or IT services) and rates of hACSC.

**Fig. 1:**
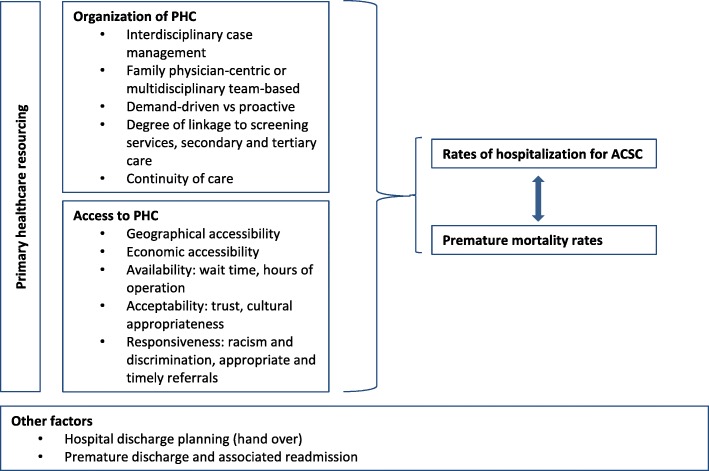
Determinants of Rates of Hospitalization for ACSC and Associated Premature Mortality Rates

With the exception of Van Loenen and colleagues [29], past research has generally not examined how the organization of the PHC can prevent such hACSC [30]. These international comparisons, however, generated contradictory results [29, 30] suggesting the importance of contextual nuancing [23]. Urban-centric work also dominated research in this field; most studies have focused on large geographical areas and aggregated data across these areas, thereby erasing the specific experience of small rural and remote communities. The few studies that focused on small populations and rural/remote analyses [4, 31–35] have shown that variability in access, quality and responsiveness are important to consider. Finally, few longitudinal studies have been conducted [4, 31, 36] to analyze trends in hACSC over time, or to document the potential impact of policy or organizational shifts on hACSC. Figure 1 summarizes known determinants.

The purpose of this paper is to examine whether hACSC: (1) can be used as a proxy measure for access to responsive PHC in First Nations reserve areas and (2) is associated to premature mortality rate (PMR). This work seems particularly relevant to studies of marginalized and vulnerable populations [4, 31, 37], where outcomes continue to be linked to differential treatment [38–41].

This work is also timely: On October 1st, 2013, the BC First Nation Health Authority (FNHA) took over a range of responsibilities previously shouldered by a federal agency, namely the First Nations and Inuit Health Branch of Health Canada (FNIHB). It is therefore important to note that all findings presented in this paper *predate* the transfer of health services to the FNHA and therefore do not reflect subsequent investments or enhancements of PHC on-reserve in British Columbia after October 1, 2013. Still, this work may help inform priority setting for the FNHA.

## Methods

The Closing the Gap study is a partnership between the First Nations Health Authority (FNHA) and University-based health researchers from the University of Manitoba, the University of British Columbia, Simon Fraser University and Queens University. Throughout this project, oversight of data interpretation and publications was provided by the FNHA to ensure that the findings were understood in context.

We conducted a secondary analysis of a linked dataset. Multilevel modeling was used in order to capture both the individual (sex, age) and community level characteristics (local access to PHC) that predict hACSC for each resident of a First Nations reserve in BC.

### Conceptual framework

In the First Nations context, on-reserve PHC services are funded (and were historically delivered) by FNIHB, whereas services for other Canadians are provided (hospitals, public health) or funded (primary care) by provincial healthcare systems. In the 1980s First Nations communities increasingly began to assume more control over community-based on-reserve health services [42]. In October 2013, the FNHA took over the funding and management of all First Nations health services on behalf of FNIHB. This new model is unprecedented in Canada, and has no equivalent internationally.

First Nation communities in Canada can range from less than 100 to over 15,000 residents. British Columbia’s 199 First Nation communities range from less than 100 to around 3500 residents, with an average of approximately 200 residents. These communities are spread across the province, a territory of just under 950,000km^2^. While many First Nation communities are located close to provincial community, many more are considered remote and/or isolated, making access to equitable care a challenge.

Table 1 shows the four-level framework used by FNIHB, and inherited by the FNHA, to fund on-reserve health services. Specific services accessible on reserve are associated with each level. Since the late 1980s, this framework has informed funding levels to communities who want to exercise greater control over their local services. Factors that determine the level of services include community size, remoteness, and accessibility of provincial services (proximity, availability of road access, quality of roads i.e. seasonal or year-long, paved or not). Communities considered to have reasonable access to provincial healthcare services in nearby communities are funded to offer screening and preventive services on a part-time basis (Health Stations, *n* = 42). Communities located within a two-hour drive from provincial services are funded to ensure local access to preventive, screening, and emergency care. These services, delivered through Health Centres (*n* = 44), focus on primary prevention, with some level of secondary prevention interventions provided by community health nurses and community staff. There is no or limited funding to ensure off-hours coverage, and no funding available for primary care. More isolated communities served by Nursing Stations (*n* = 10), are funded to ensure local access to screening, prevention, emergency and treatment services on a 24/7 basis, delivered by community health nurses and staff, and primary care nurses with an extended scope of practice. This extended scope of practice requires RNs to receive additional training and certification in remote nursing in order to meet the primary care needs specific to remote communities.

**Table 1:** Types of services available

Type of Facility	N communities	Community characteristics	N individuals (2010)	Primary healthcare dimension included			
				Primary prevention	Secondary prevention	Tertiary prevention	Primary care
Nursing Station	10	Population: Over 500 Isolation: Remote/ isolated: Over 350 km to service centre Health Services: Nearest hospital more than 2 h away, limited ambulance and first response services Transportation: No year round road access to other health care facilities Infrastructure: Limited community services Facility Capacity: local access to screening, prevention, emergency care and treatment services on a 24/7 basis. PHC delivered by primary care nurses with an expanded scope of practice, community health nurses, and paramedical staff.	3425	X	X	X	X
Health Centre	44	Population: Over 500 Isolation: Non-isolated/ semi isolated: between 50 and 350 km from service centre Health Services: Nearest hospital by road in less than 2 h; occasional unavailability of ambulance and first response services Transportation: All weather road/ air access; poor road conditions Infrastructure: Limited community services Facility Capacity: Emergency, screening and prevention available 5 days/week. There is no or limited funding to ensure off-hours coverage	8509	X	X	X	
Health Station	42	Population: 0–1000 Isolation: Remote/ isolated or semi-isolated: over 350 km from service centre but within 50 km of health centre Health Services: Nearest hospital more than 2 h away; limited ambulance and first response services Transportation: Accessible by air or road from FNIHB facility; poor road conditions Infrastructure: Limited community services Facility Capacity: Part-time, often non-resident screening and prevention services only	17,742	X	X		
No Facility	103	No on-reserve facility: access to PHC is through a provincial point of care located close to the community, and accessible through year-round roads.	13,742	X	X		

Definitions

Remote Isolated: No scheduled flights, minimal telephone or radio services, no road access

Isolated: Scheduled flights, good telephone services, no year-round road access

Semi-Isolated: Road access greater than 90 km to physician

### Cohort and First Nations identification

Our sample included all BC residents eligible under the provincial Medical Services Plan (MSP) living on First Nations reserves (estimated at 51,000 FN in BC, [43]). Consolidation File – Registry BC’s administrative data was used to track ways in which residents of First Nations communities have accessed provincial health services over time. In BC, residents must pay an additional tax (premium) dedicated to healthcare. For First Nations, this tax was paid by the federal government (prior to October 1, 2013) and tracked in the BC administrative data. As a result, we were able to use both a proxy for First Nation identification (premium payer) and six-digit postal codes to track First Nations individuals living on reserve in BC.

### Variables

A key dependent variable for this study is hACSC. We followed the recommendation of Caminal et al. ([23], p., 246) that “the [ACSC] list should be adapted to the context of each study to guarantee the validity, reliability and magnitude of the hospitalization rate; particularly when health systems are different.” We developed a definition of ACSC, which has been previously validated [4, 31]. We modified the definition based on Billings et al. [17] and the Canadian Institute of Health Information [44] and added components from the Victorian Government of Australia which is more comprehensive [45]. Table 2 shows our final definition using recent studies related to the epidemiological profile of First Nations in MB, ON and BC [5, 46–48]. Each condition was defined based on the International Classification of Diseases. We used two different measures of hospitalization: *Rates of episodic hospital care:* the discrete number of hospitalization episodes from admission to discharge. Hospitalizations were treated as a single episode when readmission to another hospital occurred within one day, to account for transfers from one hospital to another. *Rates of length of stay:* an average of the number of days in hospital for each episode of care.

**Table 2:** Definition of ACSC

	Conditions
Chronic conditions	Asthma, Angina, Heart Failure and pulmonary edema, Convulsion & Epilepsy, Diabetes with complications, Hypertension, COPD, Pneumonia, Bronchitis and Anemia
Vaccine preventable conditions	Diptheria, Hemophilus, Influenza type B, Hepatitis A, Hepatitis B, Influenza, Measles, Meningococcal disease (meningitis), Mumps, Pertussis, Pneumococcal, Poliomyelitis, Pulmonary/other, Tuberculosis, Rubella, Tetanus
Acute conditions	Dental Conditions, Cellulitis, Pelvic Inflammatory Disease, Gastroenteritis & Dehydration, Severe Ear, Nose and Throat (ENT) infections
Mental health conditions	Schizophrenia, Mood Disorders

The second key dependent variable was premature mortality rate (PMR). Premature mortality is a measure of potential years of life lost before the age of 70 years. Since the deaths of younger people are often preventable, the premature mortality rate is a measure that gives more weight to the death of younger people than to older people [49]. Our final dataset included information on hospitalizations and demographic characteristics of First Nations individuals living on-reserve in BC and community characteristics, including local access to PHC. A key independent variable explored in this study focuses on local access to PHC care.

### Sources of data

We used Discharge Abstract Database (DAD), Consolidation file, Census data (1994–2010) and Vital Stats Deaths from files held at Population Data BC [50, 51]. The data contained demographic and ecological information (such as geo-codes indicating location of residence). The DAD contains data on discharges, transfers and deaths of in-patients and day surgery patients from acute care hospitals in BC. The Consolidation file is BC’s central demographics file for research requests. It contains basic demographics such as age and sex, geo-codes indicating location of residence, and registration data. Finally, the Consolidation–Registry data files contains data on medically necessary services provided by fee-for-service practitioners to individuals covered by the Medical Services Plan (MSP), BC’s universal insurance program. It is important to note that we used the Consolidation– Registry data files to aid in the identification of First Nations participants who live on reserve.

The data source on *Community information* was obtained from a database created by Lavoie based on information in the public domain [52, 53], which contains six-digit postal code information for each on-reserve community, showing the level of care available on reserve (see Table 1); information garnered from First Nations community profiles obtained from the Aboriginal Canada Portal and other public sources; and Indigenous and Northern Affairs Canada and FNIHB on-reserve population figures. All files were linked by Population Data BC using a unique identifier created specifically for this study. All analyses used anonymized (‘de-identified’) data. All procedures were approved by the University of Manitoba (HS185005 (H2015:064) and the University of British Columbia (H11–01070) Ethics committee and access to data was approved by PopData BC’s data steward.

### Data analysis

Table 3 shows the demographic distribution of our population.

**Table 3:** Demographic distribution of population under study

Years and gender	Population FN on reserve		Population FN off reserve		Population other BC		Population All BC	
	1994	2010	1994	2010	1994	2010	1994	2010
Breakdown by sex								
Male	17,877	27,942	33,589	37,072	1,658,510	2,025,662	1,711,427	2,090,676
Female	19,328	26,527	37,169	39,708	1,668,900	2,042,029	1,723,946	2,108,264
Breakdown by age group								
0–14 yrs	11,936	12,851	22,137	15,425	694,320	655,442	728,393	683,718
15–24 yrs	6674	9845	13,147	14,036	455,899	559,747	475,720	583,628
25–34 yrs	6615	7976	14,847	12,601	568,372	585,351	589,834	605,928
35–44 yrs	5540	7404	10,431	13,096	595,123	604,277	611,094	624,777
45–54 yrs	3189	8222	5828	12,017	440,692	705,420	449,709	725,659
55–64 yrs	2067	5336	2972	6509	304,327	598,663	309,366	610,508
65–74 yrs	1184	2835	1396	3096	268,677	358,791	271,257	364,722
Breakdown by SES								
1 (lowest)	12,191	21,780	27,714	28,904	673,365	786,331	713,270	837,015
2	5006	9215	15,015	16,676	675,128	806,470	695,149	832,361
3	6337	10,808	10,982	12,428	664,022	832,889	681,341	856,125
4	8442	7295	9956	10,766	665,921	836,042	684,319	854,103
5 (highest)	5229	5371	7091	8006	648,974	805,959	661,294	819,336

We developed a multi-level model to predict hospitalization (separation and length of stay) for hACSC. We used the generalized estimating equations (GEE) method to test for differences in hospital utilization rates for hACSC. GEEs are used as a method for analyzing correlated longitudinal data. This data has measurements (hospitalization) taken over time (1994–2010) on subjects that share common characteristics (age group, sex) living in communities with similar characteristics (level of community control, access to care at the community level). Therefore, one may expect the outcomes for subjects of similar age, sex and community to be correlated over time. The GEE method reflects the correlated structure of the data and allows for valid hypothesis testing results. Measuring trends over time allow us to assess the impact of policy changes on communities over time.

Given that most individuals in any one year were not hospitalized, we used a zero-augmented beta distribution, rather than postulating normality.

## Results

Table 4 shows that at the end of the study, the adjusted PMR were higher in First Nations communities, 2006–10 (5.09) compared to all BC (2.36). While the PMR for communities served by Nursing Stations was 4.01, it was 4.64 and 4.74 for communities served by Health Stations and Health Centres, respectively.

**Table 4:** Premature mortality rates, adjusted by age, sex, and socioeconomic status 1994–1998 and 2006–2010 by facility type

Rolling 5 years	No facility	Health Station	Health Centre	Nursing Station	All facilities (all FNs in FN comm)	All other BC	All BC
1994–98	4.88	5.15	6.12	2.79	5.17	3.01	3.02
2006–10	.96	4.64	4.74	4.01	4.94	2.36	2.38

Table 5 shows there was a strong correlation between premature mortality rates and rates of hACSC in communities served by a Nursing Station (where PHC services are provided by nurses) and in other urban BC (where PHC services are generally easily accessible and provided by Family Physicians, as described in the introduction). The correlation was close to 1.0, indicating that as rates of hospitalization drop, so does the premature mortality rate. For Nursing Stations, this means that the dropin rates of hospitalization are related to healthcare needs being met. We found a similar correlation for communities with Nursing Stations and for urban BC, suggesting that having primary care provided in the community, a key feature of Nursing Stations, is key to lowering hACSC. This is particularly true for chronic conditions, where the correlations are the same (0.93 for episodes of care and 0.91 for length of stay).

**Table 5:** Correlation between directly adjusted rates of episodes of hospital care and premature mortality rates, 1994–2010

		No Facility	Health Station	Health Centre	Nursing Station	Other Rural BC	Urban BC	All Other BC	All BC
All ACSC conditions	Episodes of care	−0.53	0.24	0.64*	0.90**	0.65	0.89*	0.99**	0.99*
	Length of stay	−0.40	0.27	0.75*	0.93**	0.69	0.96**	1.0**	0.99**
Chronic conditions	Episodes of care	−0.46	0.11	0.48	0.93**	0.64	0.93**	0.99**	0.99**
	Length of stay	−0.22	0.13	0.70*	0.91**	0.75*	0.91*	0.99**	0.99**
Vaccine preventable conditions	Episodes of care	−0.5	0.26	0.69*	0.75*	0.45	0.73*	0.96**	0.96**
	Length of stay	−0.71*	0.32	0.68*	0.30	0.37	0.21	0.93**	0.92**
Acute conditions	Episodes of care	−0.53	0.24	0.66*	0.73*	0.84*	0.43	0.95**	0.95**
	Length of stay	−0.58*	0.55	0.59*	0.72*	0.73*	0.79*	0.98**	0.98**
Mental health conditions	Episodes of care	−0.28	0.33	0.63*	0.75*	0.53	0.38	0.93**	0.93**
	Length of stay	−0.24	0.28	0.28	0.77*	0.55	0.96**	0.85**	0.85**

*p < 0.05

**p < .001

Other cells = results were not statistically significant

In contrast to Nursing Stations, communities served by a Health Centre have a lower correlation (0.64 for episodes of care and 0.75 for length of stay). Based on FNIHB’s policy, Health Centres do provide prevention-oriented services, but do not offer community-based primary care (treatment) services. These services are accessed usually from Family Physicians practicing in communities located between 60 and 250 km from the reserve.

We found no correlation between hACSC and premature mortality rates in communities served by Health Stations and in communities with no facility, where residents are expected to go off reserve to access all care.

## Discussion

Results from this longitudinal study provide evidence that hACSC can be used as a proxy measure for access to PHC in for First Nations peoples living reserve areas. We suggest that in addition, hACSC could be used as an indicator to measure equity in access to responsive PHC in rural and remote communities. Indeed, our results show a strong correlation between hACSC and premature mortality rate in rural and remote on-reserve communities. We suggest this is an extremely important finding for rural and remote communities whose needs have historically been overshadowed by urban-centric data and where context-relevant evidence is badly needed in order to improve outcomes.

Using hACSC as an indicator, our findings show that Nursing Stations in remote on-reserve communities (and located at a significant distance from other providers of PHC) appear to be providing services nearing PHC services available in urban BC communities. This suggests that in remote on-reserve communities a Nursing Station-like level of services, where local access to PHC is primarily provided by nurses with an expanded scope of practice, may be better equipped to meet PHC needs than the Health Centres and Health Stations we studied. Understandably, local services available across communities are likely to vary and thus more work is needed to examine where Nursing Stations and other communities can learn from each other about aspects of the care that are promising practices. However, these results are similar to what was found in Manitoba, where on-reserve services operate on a framework similar to that used in BC [4]. Therefore, wider integration of resident RNs who have a relationship with community members, and of mechanisms where community members can help shape the service provided, into the organization and delivery of PHC could help to strengthen PHC.

Significant differences remain in the rates of hACSC for First Nations compared to all BC, suggesting that improvements are needed in ensuring responsive, culturally safe and integrated models of care. This will require increased investment, innovation, improvements and enhanced integration of First Nations cultural knowledge and participation in the health care system. First Nations in BC have given this mandate to the FNHA, where such efforts are already underway.

We recognize that this study is observational and as such our results can only document associations. Although there is a temporal element in the predictor-outcome relationship, causal inferences are still somewhat disputable. However, longitudinal studies permit more reliable prediction by borrowing information from all individuals to better predict within-individual change over time. We are using broad categories for on-reserve PHC, which gloss over the variability of services delivered on-reserve. Still, we believe that the approach we developed with the FNHA is the most pragmatic and appropriate method to provide the FNHA a baseline to inform decision-making. A second limitation of this methodology is that hospitalization rates for ACSC reflect the variability in hospitalization criteria, within and between hospitals, as well as healthcare staff decisions [23]. Thirdly, we recognize that our analysis hinges on geocoding where First Nations living on-reserve access primary care. While it is reasonable to assume that First Nations living in remote and remote isolated communities (which are generally served by nursing station or health centre) access primary care primarily on reserve, our experience suggests that residents of semi-isolated and non-isolated communities are more likely to access primary care from a variety of source. We therefore anticipate that our results are less robust for communities served by health offices. Communities with no facility on-reserve are by definition receiving care off-reserve. Finally, we cannot identify all First Nations individuals living on reserve, since the premium paid by the Federal government is only for those who are registered as “status” Indian (i.e those who are recognized as Indians and therefore entitled to specific rights under the Canadian constitution). However, 91.4% of First Nations people living on reserve in BC are status First Nations.

## Conclusions

This study adds important findings to a small body of work examining PHC in First Nations communities rural and remote communities. As a proxy measure, hACSC could be considered an indicator of equity in access to PHC in these communities. Moreover, in the absence of data collected from each on-reserve community, hACSC could be used as a proxy measure for the responsiveness of PHC in these communities. In the communities included in this study, PHC was primarily provided by accessing services provided by community health staff and nurses with an expanded scope of practice, and supplemented with off-reserve services as needed.

While our results confirm that local access to PHC results in better outcomes, especially in Nursing Stations, it is likely that a single solution to improve access to PHC for all First Nations in BC will not fit all. Localized solutions, developed in partnership between First Nations communities, the FNHA and the Regional Health Authority are needed. More work is required to understand why local access to a complement of PHC that includes primary care is significant. Possible factors include greater integration between PHC and primary care, when provided by a single team on reserve; better integration of local context in care plans; and better integration with other health services provided on reserve. Additional research to identify which, if any, of these factors may be at play would be helpful.

The analysis we present provides (1) a potential baseline that the FNHA can use in the future to evaluate changes in access to responsive PHC; and (2) direction as to methods the FNHA may utilize in the future to support decision-making. We acknowledge that First Nations communities in BC are diverse, with some located in areas with good access to responsive PHC, some located in remote isolated regions where PHC can be accessed on reserve, and others experiencing considerable challenges accessing limited PHC delivered by Family Physicians off reserve because of road conditions (logging roads, winter conditions). Further, the Regional Health Authorities (RHAs) have historically developed different relationships with First Nations communities located within their catchment areas, with some acknowledging a responsibility to improve access to PHC on reserve, and others having a somewhat less proactive approach. This was particularly true during the period under study (1990–2010). The organization of hospital-based care and PHC also varies in regions, with access to hospital-based acute care being consolidated to larger centres, complemented in some regions by a limited number of smaller community-based hospitals offering limited services. In addition, access to family physicians in BC’s rural and remote communities remains highly variable, and continuity of care is often compromised by turnover and gaps in coverage. Finally, there was a fundamental shift in 2013 following the creation of the FNHA. As a result, the FNHA has since been able to effectively advocate for: shared decision-making in regional planning; the better integration of provincially-provided health services with those provided on reserve; and the integration of First Nation concepts of health and wellness in provincial program delivery [54]. For these reasons we cannot advocate for a single solution to improve access to PHC, even though our data confirms that local access to PHC results in better outcomes. Instead, we recommend localized solutions developed in partnership between First Nations communities, the FNHA and the RHAs.

While the role of PHC in mitigating social exclusion, discrimination, and racism is limited, access to effective and responsive PHC services can be an important lever in softening their impacts and improving outcomes. A recent study by Browne and colleagues [55] demonstrated that there are key dimensions of effective equity-oriented PHC. They argue that the delivery of these key dimensions of care required four complementary approaches: developing partnerships with Indigenous peoples, taking action at all levels, paying attention to local and global histories, and attending to the unintended and potentially harmful consequences of each strategy [56]. We add that for rural and remote environments, effective equity-oriented PHC also includes access (direct and facilitated) to hospital and ongoing specialist care off reserve or via telehealth.

## Abbreviations

ACSC: Ambulatory care sensitive conditions
BC: British Columbia
COPD: Chronic obstructive pulmonary disease
DAD: Discharge Abstract Database
FN: First Nations
FNHA: First Nations Health Authority
FNIHB: First Nations and Inuit Health Branch of Health Canada
GEE: Generalized estimating equations
hACSC: Hospitalizations for ambulatory care sensitive conditions
MB: Manitoba
MSP: Medical Services Plan
ON: Ontario
PHC: Primary health care
PMR: Premature mortality rate
UBC: University of British Columbia
